# Association between Serum Vitamin D and Metabolic Syndrome in a Sample of Adults in Lebanon

**DOI:** 10.3390/nu15051129

**Published:** 2023-02-23

**Authors:** Myriam Abboud, Rana Rizk, Suzan Haidar, Nadine Mahboub, Dimitrios Papandreou

**Affiliations:** 1 Department of Health, Zayed University, Abu Dhabi P.O. Box 144534, United Arab Emirates; 2 Department of Natural Sciences, School of Arts and Sciences, Lebanese American University, Byblos P.O. Box 36, Lebanon; 3 Institut National de Santé Publique, d’Epidémiologie Clinique et de Toxicologie-Liban (INSPECT-LB), Beirut, Lebanon; 4 Department of Nutrition and Food Sciences, Lebanese International University, Beirut P.O. Box 146404, Lebanon

**Keywords:** metabolic syndrome, vitamin D, Lebanon, adults

## Abstract

The evidence on the association between vitamin D and metabolic syndrome (MetS) is inconclusive. This was a cross-sectional study to explore the relationship between vitamin D serum levels and MetS in a sample of Lebanese adults (*n* = 230), free of diseases that affect vitamin D metabolism, recruited from an urban large university and neighboring community. MetS was diagnosed according to the International Diabetes Federation criteria. A logistic regression analysis was performed taking MetS as the dependent variable, and vitamin D was forced into the model as an independent variable. The covariates included sociodemographic, dietary, and lifestyle variables. The mean (SD) serum vitamin D was 17.53 (12.40) ng/mL, and the prevalence of MetS was 44.3%. Serum vitamin D was not associated with MetS (OR = 0.99 (95% CI: 0.96, 1.02), *p* < 0.757), whereas the male sex, compared with the female sex and older age, was associated with higher odds of having MetS (OR = 5.92 (95% CI: 2.44, 14.33), *p* < 0.001 and OR = 1.08 (95% CI: 1.04, 1.11), *p* < 0.001, respectively). This result adds to the controversy in this field. Future interventional studies are warranted to better understand the relationship between vitamin D and MetS and metabolic abnormalities.

## 1. Introduction

Metabolic syndrome (MetS) is a constellation of metabolic derangements which includes abdominal obesity, increased fasting blood glucose (FBG), elevated blood pressure (BP), elevated triglycerides, and low high-density lipoprotein cholesterol (HDL-C). MetS is strongly associated with increased morbidity, mortality [1], and healthcare costs [2]. Globally, MetS prevalence ranges between 10 and 84%, depending on the definition used, sex, race, and geographical distribution of the population under study [2,3]. The primary approach in its management is to modify underlying environmental risk factors, including excessive body weight, sedentary lifestyle, atherogenic diet, smoking, and alcohol consumption [4,5,6].

Vitamin D is a fat-soluble prohormone, which was previously associated with bone mineral metabolism [7]. Over time, extra skeletal functions of vitamin D have been suggested, and recently, there has been a growing number of studies linking vitamin D insufficiency to MetS and its components. This association was suggested due to largely overlapping risk factors, such as inadequate exercise and lack of sun exposure [8]. Studies have shown that a lack of vitamin D lowers intracellular calcium levels, preventing cells from releasing insulin, thus decreasing glucose tolerance. Furthermore, vitamin D increases the number of insulin receptors, which are crucial for insulin responsiveness and glucose metabolism. Moreover, vitamin D possesses hormonal, anti-inflammatory, anti-apoptotic, and anti-fibrotic properties, suggestive of its MetS-preventive properties [7]. 

Vitamin D is typically measured through serum levels of 25-hydroxyvitamin D (25(OH)D). Observational data have revealed that a rise in blood 25(OH)D levels by just 1 ng/mL was associated with a 54% lower risk of MetS, whereby a serum 25(OH)D level was associated with atherogenic dyslipidemia [9]. However, some studies found no association between vitamin D serum levels and MetS in adults [10,11,12]. In a recent systematic review, observational data showed a strong correlation between vitamin D and components of MetS, i.e., obesity, dyslipidemia, blood pressure, insulin, and glucose metabolism, and experimental data indicated a positive effect of vitamin D supplementation on blood pressure, abdominal obesity, insulin, and glucose metabolism [13]. Another recent systematic review and dose-response meta-analysis showed that, in cross-sectional studies and cohort studies, respectively, a 10 ng/mL increase in vitamin D concentration was linked to 20 and 15% decreased chances of MetS [14].

Despite being a sun-rich country, vitamin D deficiency is frequently observed in Lebanon. Around 50% of older persons and 72.8% of adults have serum vitamin D levels below 10 and 12 ng/mL, respectively [15,16]. Moreover, MetS is also widespread in the country, with estimates ranging between 23.5 and 31.2% [17,18]. Two previous studies examined the association between vitamin D and MetS among specific populations in Lebanon. Ghadieh et al. [18] showed that among employees of one private university, those with vitamin D < 20 ng/mL had 2.5 higher odds of having MetS than those with adequate vitamin D. Moreover, among the components of MetS, only hypertriglyceridemia and low HDL were associated with inadequate vitamin D. Gannge-Yared et al. [19] explored the association between vitamin D and each of the components of MetS among non-obese students with adequate vitamin D status recruited from one private university and reported a significant association with FBG only. Due to the limited evidence on the association between MetS and vitamin D, the aim of the present study is to examine this association among Lebanese adults.

## 2. Materials and Methods

### 2.1. Design

This study of cross-sectional design was conducted in a sample of Lebanese adults during May 2022. Subjects were recruited from a large university and neighboring community via community announcements. 

### 2.2. Subjects

Participants were requested to come to fast for more than 8 h on the day of data collection and were only included in the study if they were between 18 and 65 years of age, Lebanese, neither pregnant nor lactating, not using medications affecting vitamin D metabolism, such as those taking seizure and antituberculosis drugs, and free of diseases affecting vitamin D metabolism, as in the case of severe renal or liver disease, and free of active infections such as COVID-19 [20]. 

### 2.3. Ethical Considerations

Trained research assistants confirmed eligibility and informed participants about the study protocol. All participants consented before data collection. The study was ethically approved by the Lebanese International University’s Institutional Review Board (IRB) (case number: LIUIRB-220201-SH-111).

### 2.4. Data Collection

#### 2.4.1. Blood Sample

After taking a seated resting position for at least five minutes, a 5-mL blood sample was taken from the participants by a phlebotomist into a sterile serum separator tube with a clot activator. Samples were transported to the laboratory using a thermally insulated box, where they were centrifuged at 4000 revolutions/minute for ten minutes and analyzed for total cholesterol (mg/dL), HDL-C (mg/dL), triglycerides (mg/dL), FBG (mg/dL), and 25(OH)D (ng/mL) using an automated chemiluminescence micro-particle immunoassay (CMIA) kit (ARCHITECT; Abbott Laboratories, Abbott Park, IL, USA). For the present study, the vitamin D deficiency cut-off was 20 ng/mL [21].

#### 2.4.2. Blood Pressure

A nurse measured BP using a standardized mercury sphygmomanometer following best practice. Two consecutive readings on the same arm were recorded, and their average was used for analysis [22].

#### 2.4.3. Anthropometry

Trained dietitians collected the anthropometric data from the participants using standardized techniques and calibrated equipment. Height (cm) and weight (kg) were measured using a portable stadiometer (ADE, Germany) and a beam scale, respectively. Participants took their shoes and heavy clothes off. Height was taken to the nearest 0.1 cm, and weight to the nearest 100 g while the head was positioned in the Frankfort plane. Body mass index (BMI) was calculated as the ratio of weight (kg) and height squared (m^2^). Waist circumference (to the nearest 0.1 cm) was taken via a measuring tape at the midpoint between the right iliac crest and the lower costal region [23].

#### 2.4.4. Diagnosis of Metabolic Syndrome

The International Diabetes Federation (IDF) criteria [24] were used to diagnose MetS. Participants were considered to be suffering from MetS if they had central obesity (≥94 cm in males and ≥80 cm in females; or BMI > 30 kg/m^2^, thus assuming central obesity) and two of the following factors: elevated triglycerides (≥150 mg/dL) or being treated for it; low HDL-C (<40 mg/dL in males and <50 mg/dL in females) or being treated for it; raised BP (systolic BP ≥ 130 or diastolic BP ≥ 85 mmHg) or treatment for hypertension; and FBG ≥ 100 mg/dL or diagnosed type 2 diabetes.

#### 2.4.5. Questionnaires

The following questionnaires were used and filled out by trained research assistants to limit ambiguity:

*Demographic and medical history questionnaire:* includes questions about the age, sex, education, employment and socioeconomic status, smoking status, and history of chronic diseases. 

*Mediterranean Diet Adherence Screener (MEDAS):* Taken from the Prevencion con Dietamediterranea (PREDIMED) and translated into Arabic [25]. The screener includes 14 questions pertaining to food intake and frequency of food/food ingredients. When an answer to a question is in favor of the Mediterranean diet pattern, one point is scored, whereas unfavorable responses are assigned a 0 score. The final value, calculated by adding all question scores, ranges between 0 and 14, whereby higher values denote a greater adherence to the Mediterranean diet. 

*The International Physical Activity Questionnaire (IPAQ)—Short Form* [26]: Includes seven questions aimed to identify both duration and frequency of physical activity performed in the past week. Metabolic equivalent of tasks (METs) are calculated by multiplying the total minutes expended in a certain activity by the frequency (days) by the constants of 3.3 for light, 4.0 for moderate, and 8.0 for vigorous activity. Total METs are the sum of the respective MET values for activities performed for more than 10 min. The Arabic version of the IPAQ—Short Form was used [27].

*The Pittsburgh Sleep Quality Index (PSQI):* Includes nine questions addressing sleep quantity and quality. The total score is calculated, with higher scores (≥5) indicating poor sleep [28]. The Arabic version, which was culturally adapted by Haidar et al. [29], was used.

*The 10-item Cohen Perceived Stress Scale (PSS-10):* A ten-item questionnaire assessing the levels of stress in the last month [30]. PSS uses a 5-point Likert scale ranging between never (0) and very often (4). Final scores range between 0 and 40, with higher scores indicating higher levels of perceived stress. The Arabic version, which was validated by Chaaya et al. [31], was used.

*The modified Yale Food Addiction Scale (mYFAS-Ar-Leb):* This is the Arabic version to diagnose food addiction [32]. This is a nine-item questionnaire, including one item from each of the symptom groups that compose the seven diagnostic criteria for substance use disorders of the DSM-4 Text Revision [33], plus two individual items assessing clinical impairment and distress. Food addiction is diagnosed when a person endorses at least three dependence symptoms and meets the criterion for clinical significance. The mYFAS-Ar-Leb is a validated tool in the Lebanese population. 

Before data collection, the survey was pilot-tested on ten adults, based on which the final version was produced.

### 2.5. Sample Size Calculation

We used Epi-info version 7.2 to calculate the minimum sample size, with a 1 − β = 0.8 and a 95% confidence level. The outcome percentage was retrieved from a Lebanese study evaluating the association between vitamin D and MetS, where 52.3% of those with inadequate vitamin D level had MetS, and an odds ratio of being diagnosed with MetS of 2.5 among those with inadequate vitamin D was reported [18]. It was determined that 184 participants were required, for which we added 10% (*n* = 19) to account for missing data, leading to a target of 204 participants.

### 2.6. Statistical Analysis

SPSS (version 25) was used to analyze the data. Counts and percentages were used to summarize categorical variables and mean and standard deviation for continuous measures. The bivariate analysis included chi-square and Fisher exact tests to compare categorical variables and the Student t-test to compare means of two groups. A logistic regression analysis was then conducted using the Enter method, taking MetS as the dependent variable and vitamin D serum level and lifestyle variables as the independent variables adjusted over the sociodemographic variables. All covariates showing a *p* < 0.2 in the bivariate analysis were entered in the model, while vitamin D was forced. Statistical significance was determined with a *p*-value < 0.05.

## 3. Results

### 3.1. Demographics and Medical Characteristics

The mean age of the sample was 43.36 ± 16.05 years. The majority of study participants were females (62.9%) and married (55.6%). More than half of study participants (53.8%) had a high school education or below, and 53% were unemployed. Just over a quarter were cigarette smokers (28.5%), and 41.6% were water-pipe smokers. More than half of the sample had a family history of diabetes (54.8%) or hypertension (57.3%), and 38.6% had a history of dyslipidemia. The majority had no food addiction (81%). The total mean IPAQ (log10) score among the study population was 3.15 ± 0.49, indicating moderate physical activity. The mean total PSQI was 6.99 ± 3.63; a score ≥ 5 indicates poor sleep quality. The average PSS was 19.84 ± 7.32, denoting moderate stress levels in the previous month. The mean MEDAS score was 5.98 ± 2.17, suggesting low adherence to the Mediterranean diet. The mean 25(OH)D level was 17.53 ± 12.40 ng/mL, denoting vitamin D deficiency. As for the prevalence of MetS, 44.3% (*n* = 98) of the sample were diagnosed with it.

The association of sociodemographic and lifestyle factors with MetS is shown in Table 1. Significant differences were observed in sociodemographic characteristics between study participants who had MetS and those who did not, including gender, age, education level, and marital status (*p* < 0.05). The association between MetS and cigarette smoking showed borderline significance (*p* = 0.05), whereby 68.8% of smokers and 51.1% of current smokers had MetS compared with 39.9% of nonsmokers. With regards to lifestyle characteristics and vitamin D serum levels, none were found to be significantly different by MetS status (*p* > 0.05).

### 3.2. Association between Vitamin D and Metabolic Syndrome

Table 2 presents the logistic regression for MetS and vitamin D status, adjusting for numerous sociodemographic and lifestyle factors. The results showed that vitamin D was not associated with MetS (OR = 0.99 (95% CI: 0.96, 1.02), *p* < 0.757). In contrast, the odds of having MetS, were approximately six times greater among males compared with females (OR = 5.92 (95% CI: 2.44, 14.33), *p* < 0.001). In addition, older age was associated with higher odds of MetS (OR = 1.08 (95% CI: 1.04, 1.11), *p* < 0.001).

## 4. Discussion

This study explored the relationship between vitamin D and MetS. The logistic regression models revealed that the participants’ vitamin D level was not associated with MetS. Furthermore, the present study found alarming prevalence rates of MetS (44.3%), which were positively associated with older age and the male gender. 

### Association between Vitamin D and MetS in Study Population

In this study, serum vitamin D levels were not statistically associated with MetS after adjusting for age, sex, and lifestyle factors (physical activity, sleep, stress, food addiction, and smoking). Participants with MetS had slightly higher serum levels of vitamin D in comparison with those without MetS; however, this association was not significant. 

The evidence surrounding vitamin D and MetS association is inconsistent. Some studies revealed an inverse relationship [18,34,35], while others did not report such an association [19,35,36,37,38,39]. Additionally, it is uncertain which components of MetS might be involved in this association, with some studies suggesting obesity, while others suggest glucose homeostasis.

The contradictory findings from this study may be due to several factors. First, differences in the general study characteristics, including the study design, definition of MetS, unit and method of measuring serum vitamin D, and the representativeness, size, and health status of the sample, in addition to adjustments for confounders, might impact the findings of these studies. Second, factors such as age, sex, socioeconomic status, pregnancy, clothing style, sun exposure, seasons, latitude, pollution, BMI, and skin pigmentation might affect serum vitamin D levels [40,41]. Genetics might also affect vitamin D status [7]; for instance, the difference in gene expression in vitamin D-metabolizing enzymes and impaired hepatic 25-hydroxylation could result in vitamin D deficiency. Much of these factors were assessed in our study; however, other factors may be implicated in the relationship between vitamin D levels and MetS that could not have possibly been accounted for, such as sun exposure, clothing style, and genetic predisposition, etc. Likewise, Gannagé-Yared et al. [19] found that vitamin D levels are similar between subjects with and without MetS (28.65 ± 15 vs. 31.1 ± 12.34 ng/mL, respectively, *p* = 0.38). In addition, the correlation between vitamin D levels and the number of MetS risk factors was not significant (*p* = 0.09). 

The present study population is relatively small and considered a low-risk group, especially as 63% of our sample are females, 71% are non-smokers, 58% are non-waterpipe smokers, 58% are physically active, the median age is 43 years, and the participants had average stress levels. This might have hindered us from observing a significant association between vitamin D and MetS.

This study revealed that vitamin D deficiency is highly prevalent among Lebanese adults. Our results align with those reported by Chakhtoura et al. [41], whereby the prevalence of hypovitaminosis D, i.e., 25(OH)D levels below 20 ng/mL, ranged between 44 and 96%, and the mean 25(OH)D was between 11 and 20 ng/mL. The possible reasons for the widespread vitamin D deficiency in Lebanon can be related to increased obesity rates in Lebanese adults, the dietary transition to Westernized dietary patterns, and diets high in total fat, saturated fat, and sugar but low in micronutrients such as vitamin A, vitamin D, and folic acid as well as iron, calcium, and zinc, despite having long sunny days throughout the year. Moreover, Arabi et al. [40] found a high prevalence of vitamin D deficiency (39.1% using the Institute of Medicine threshold) in rural and urban areas of Lebanon in the year 2000. Likewise, Gannagé-Yared et al. [19] reported high vitamin D deficiency in a similar age group (30 to 50 years), whereby 75% of the study population were vitamin D-deficient using the same threshold. It is thus necessary to tackle vitamin D deficiency in Lebanon through evidence-based approaches.

In this study, MetS was diagnosed in 44.3% of participants; this is higher than other studies among national samples of Lebanese adults of 36 [42], 31.2 [17], and 34.6% [43]. In addition, MetS was more common in males than females. Although some studies did not report sex differences in MetS prevalence [44,45], others reported a higher prevalence of MetS among males, and others found a higher prevalence in females [46]. These conflicting results may be attributed to differences associated with physiological factors, socio-economic status, and lifestyle factors.

In our study, advanced age and being male independently increased the odds of MetS. Comparable with our results, age was positively associated with MetS across sexes; this is in line with previous studies [17,18]. Age is a risk factor for MetS. A plausible explanation may be related to the fact that with age, blood vessels progressively lose elasticity and increase their resistance, which slows blood flow. Additionally, poor circulation makes it more likely for fat to build up in the abdomen and release free fatty acids into the blood, thus increasing insulin resistance and triglyceride levels in the blood and, ultimately, the risk of MetS [47]. In addition, this could be attributed to age-related decline in numerous physiological variables and the unhealthy lifestyles adopted throughout life, which greatly increase metabolic risk factors. 

This is one of the few studies exploring the relationship between MetS and vitamin D among Lebanese adults while accounting for several confounders. A biochemical analysis was performed in a certified laboratory in Lebanon, hence ensuring high quality and validity. Moreover, validated assessment tools were used. However, the results of the study should be interpreted in the light of some limitations. First, the present study included a convenient sample of Lebanese adults, and the results may not be generalized. Nevertheless, the study sample included participants from different socioeconomic characteristics (e.g., education levels and employment). Second, as for the Lebanese population [40], the majority of the sample was vitamin D-deficient. This could potentially underestimate or confound the association with MetS. Third, this was a cross-sectional study, hence disabling causal inferences [48].

## 5. Conclusions

The study did not find an association between vitamin D status and MetS in a sample of Lebanese adults. Vitamin D deficiency was widespread in the study sample. MetS was also widespread, with higher age and the male gender being main determinants. Further studies should examine the relationship between vitamin D and MetS and its components, adjusting for a wide range of socioeconomic (income, area of residence), lifestyle (e.g., diet, alcohol), and metabolic factors (e.g., insulin resistance, body fat percentage) among Lebanese adults. Future interventional studies are warranted to confirm the causal relationship between vitamin D and MetS and metabolic abnormalities. Finally, given the widespread prevalence of vitamin D deficiency, we suggest that urgent action be taken at a national level to address the problem and prevent associated complications.

## Figures and Tables

**Table 1:** Sociodemographic and medical characteristics of the participants (N = 230).

Variable	Total (%)	Metabolic Syndrome		*p*-Value
		Yes (N = 98 (44.3%))	No (N = 123 (55.7%))	
Gender				
Male	82 (37.1%)	49 (59.8%)	33 (40.2%)	<0.001
Female	139 (62.9%)	49 (35.3%)	90 (64.7%)	
Marital status				
Single/widowed/divorced	98 (44.3%)	32 (32.7%)	66 (67.3%)	0.002
Married	123 (55.7%)	66 (53.7%)	57 (46.3%)	
Education level				
University degree	102 (46.2%)	32 (31.4%)	70 (68.6%)	0.008
High school	41 (18.6%)	21 (51.2%)	20 (48.8%)	
Middle education	37 (16.7%)	20 (54.1%)	17 (45.9%)	
Primary education	30 (13.6%)	18 (60.0%)	12 (40.0%)	
Illiterate	11 (5.0%)	7 (63.6%)	4 (36.4%)	
Employment				
Yes	103 (47.0%)	47 (45.6%)	56 (54.4%)	0.707
No	116 (53.0%)	50 (43.1%)	66 (56.9%)	
Cigarette smoking				
Never	158 (71.5%)	63 (39.9%)	95 (60.1%)	0.050
Previous smoker	16 (7.2%)	11 (68.8%)	5 (31.3%)	
Smoker	47 (21.3%)	24 (51.1%)	23 (48.9%)	
Waterpipe smoking				
Never	129 (58.4%)	60 (46.5%)	69 (53.5%)	0.673
Previous smoker	22 (10.0%)	10 (45.5%)	12 (54.5%)	
Smoker	70 (31.7%)	28 (40.0%)	42 (60.0%)	
Family history of diabetes				
No	84 (38.0%)	35 (41.7%)	49 (58.3%)	0.370
Yes	121 (54.8%)	58 (47.9%)	63 (52.1%)	
Do not know	16 (7.2%)	5 (31.3%)	11 (68.8%)	
Family history of dyslipidemia				
Yes	85 (38.6%)	42 (40.0%)	63 (60.0%)	0.266
No	105 (47.7%)	38 (44.7%)	47 (55.3%)	
Do not know	30 (13.6%)	17 (56.7%)	13 (43.3%)	
Family history of hypertension				
Yes	126 (57.3%)	31 (40.8%)	45 (59.2%)	0.633
No	76 (34.5%)	59 (46.8%)	67 (53.2%)	
Do not know	18 (8.2%)	7 (38.9%)	11 (61.1%)	
Food addiction status (mYFAS)				
Yes	42 (19.0%)	23 (54.8%)	19 (45.2%)	0.131
No	179 (81.0%)	75 (41.9%)	104 (58.1%)	
Physical activity				
Low	91 (41.4%)	49 (53.8%)	42 (46.2%)	0.030
Moderate	67 (30.5%)	29 (43.3%)	38 (56.7%)	
High	62 (28.2%)	20 (32.3%)	42 (67.7%)	
	Mean ± SD	Mean ± SD	Mean ± SD	
Age (years)	43.36 ± 16.05	52.24 ± 12.86	36.36 ± 14.84	<0.001
Vitamin D (ng/mL)	17.53 ± 12.40	17.74 ± 10.01	17.36 ± 14.05	0.819
PSQI	6.99 ± 3.63	7.48 ± 3.67	6.60 ± 3.57	0.071
PSS	19.84 ± 7.32	20.19 ± 7.48	19.56 ± 7.21	0.525
MEDAS	5.98 ± 2.17	6.11 ± 1.75	5.87 ± 2.46	0.428

IPAQ: International Physical Activity Questionnaires, PSQI: Pittsburgh Sleep Quality Index, PSS: Perceived Stress Scale, MEDAS: Mediterranean Diet Adherence Score, YFAS: Yale Food Addiction Scale.

**Table 2:** Logistic regression taking the metabolic syndrome as the dependent variable.

	*p*-Value	ORa	95% Confidence Interval	
			Lower	Upper
Gender (male vs. female *)	<0.001	5.921	2.446	14.335
Age	<0.001	1.082	1.048	1.116
Marital status (married vs. single *)	0.727	0.867	0.390	1.930
Education level (primary vs. never *)	0.328	0.391	0.060	2.561
Education level (elementary vs. never *)	0.090	0.201	0.031	1.286
Education level (secondary vs. never *)	0.267	0.359	0.059	2.192
Education level (university vs. never *)	0.054	0.176	0.030	1.029
IPAQ (Log 10)	0.793	0.902	0.419	1.944
PSQI	0.218	1.073	0.959	1.201
YFAS (yes vs. no *)	0.090	2.413	0.871	6.688
Vitamin D	0.757	0.995	0.965	1.027
Cigarette smoking (previous vs. never *)	0.207	2.972	0.547	16.154
Cigarette smoking (current vs. never *)	0.436	0.713	0.304	1.673

ORa: adjusted odds ratio, IPAQ: International Physical Activity Questionnaires, PSQI: Pittsburgh Sleep Quality Index, PSS: Perceived Stress Scale, MEDAS: Mediterranean Diet Adherence Score, YFAS: Yale Food Addiction Scale.Variables entered into the model: gender, age, marital status, education level, cigarette smoking, IPAQ, PSQI, YFAS, and vitamin D. * Reference group. *p*-values marked in bold are < 0.05.

## Data Availability

Not applicable.
